# PALOT: Profiling and Authenticating Users Leveraging Internet of Things

**DOI:** 10.3390/s19122832

**Published:** 2019-06-25

**Authors:** Pantaleone Nespoli, Mattia Zago, Alberto Huertas Celdrán, Manuel Gil Pérez, Félix Gómez Mármol, Félix J. García Clemente

**Affiliations:** 1 Department of Information and Communications Engineering, University of Murcia, 30100 Murcia, Spain; mgilperez@um.es (M.G.P.); felixgm@um.es (F.G.M.); 2 Telecommunication Software & Systems Group, Waterford Institute of Technology, X91 K0EK Waterford, Ireland; alberto.huertas@um.es; 3 Department of Computer Engineering and Technology, University of Murcia, 30100 Murcia, Spain; fgarcia@um.es

**Keywords:** continuous authentication, IoT, authorization, security, behavioral patterns discovery, Markov Model

## Abstract

Continuous authentication was introduced to propose novel mechanisms to validate users’ identity and address the problems and limitations exposed by traditional techniques. However, this methodology poses several challenges that remain unsolved. In this paper, we present a novel framework, PALOT, that leverages IoT to provide context-aware, continuous and non-intrusive authentication and authorization services. To this end, we propose a formal information system model based on ontologies, representing the main source of knowledge of our framework. Furthermore, to recognize users’ behavioral patterns within the IoT ecosystem, we introduced a new module called “confidence manager”. The module is then integrated into an extended version of our early framework architecture, IoTCAF, which is consequently adapted to include the above-mentioned component. Exhaustive experiments demonstrated the efficacy, feasibility and scalability of the proposed solution.

## 1. Introduction

The constant evolution of modern computer systems is undoubtedly changing our lives. Nowadays, they are not only effectively smaller, faster, and easier to use than before, but also they are cheaper and more pervasive. Despite their benefits, there are still some critical aspects that require more efforts and research to evolve as expected [1]. Among them, we highlight the security of the devices [2] and, specifically, the associated authentication mechanisms.

Traditional authentication systems are based on the following three well-known Authentication Factors (AFs) to identify users: some secret that a user knows, some token that a user has, or something that a user is. One of the greatest improvements of modern authentication systems is the combination of the previous three AFs in different ways, such as the two-factor authentication system involving two independent channels for authenticating the user (e.g., a smart-card and a PIN code). However, these systems still have limitations due to their nature, specifically:

- AFs that leverage on user’s knowledge such as passwords or PIN codes.
  Even if the user’s secret is not trivial to guess and it is safely stored, it remains vulnerable to social engineering attacks. On top of that, there will always be the possibility that the user may forget his authentication secret. Thus, the general attitude of the user is to choose fairly guessable, and therefore weak, passwords.
- AFs that leverage on user’s possessions such as smart-cards.
  Limitations are similar to the previous category , i.e., a physical object can be forgotten or stolen. Additionally, since the users are forced to carry around specific authentication token(s), the overall usability of the system decreases.
- *AFs that leverage on user’s intrinsic characteristics such as fingerprint or face recognition*.
  The strong aspects of this category also represent their major limitations since biometric measures cannot be lost or changed and thus they cannot be revoked or updated. Moreover, some of the devices which are in charge of measuring user’s characteristics are often intrusive and expensive, such as brainwaves or heart-rate sensors.

Not surprisingly, the continuous authentication paradigm has been increasingly employed at every level [3] with the goal of enhancing the overall security of the authentication process. Continuous authentication, also known as permanent authentication, was introduced to propose novel mechanisms to validate users’ identity, addressing the problems shown by traditional techniques [4]. This methodology can continuously authenticate the legitimacy of a user over the time by analyzing their behavioral profile, e.g., by identifying the users through their interaction with a specific device [5,6]. However, despite their numerous advantages, existing solutions based on continuous authentication still present some challenges that will characterize future studies, such as their necessity of carrying out particular devices, vulnerability to particular impersonation attacks, accuracy of the authentication process, elapsed time, and processing complexity [7].

In this authentication context, a cutting edge technology such as the Internet of Things (IoT) is starting to be considered by the academia and industry as a key driver to improve those previous challenges [8]. In fact, IoT is considered to be a critical part of the Internet of the future, where billions of “intelligent objects” will communicate to provide services to humans [9]. These devices are going to be located almost everywhere, from vehicles to buildings, home appliances, or cell phones, passively sensing the environment to collect relevant information [10]. In this context, we state that the versatility and ubiquity of IoT devices can be used to build a global, pervasive, and continuous authentication system.

By gathering, combining, and correlating events generated by different IoT devices, it is possible to create accurate behavioral profiles based on the interactions of users with the surrounding smart objects during the time. In a nutshell, the use of the IoT as a continuous authentication mechanism could improve some of the major limitations of existing authentication solutions [11]. However, at this point, the use of the IoT for authentication purposes is not mature enough and several open challenges should be addressed to demonstrate its usefulness. Among them, we highlight the management and correlation of heterogeneous events coming from different devices in real time, the use of events to create precise users’ behavior profiles, and the feasibility of resource-constrained IoT devices to run authentication mechanisms.

To tackle the previous open challenges, the main contributions of this article, an extended version of [12], are the following ones:

- A confidence manager module, fully integrated in our continuous authentication architecture, focused on recognizing users’ behavioral patterns from series of events in real time. The proposed module is able to detect behavioral patterns of users by considering sequences of events generated during the interactions of users and heterogeneous IoT devices.
- An updated formal definition of our previous information model [12], which is focused on modeling different events generated by heterogeneous devices existing in the IoT.
- The proposal of our Profiling and Authenticating users Leveraging internet Of Things (PALOT) architecture, capable of providing a continuous and non-intrusive authentication and authorization solution for users according to their interaction with the surrounding IoT devices.
- A pool of experiments that demonstrates the usefulness of our continuous authentication system to authenticate a person in an IoT-enabled environment (smart home). The proposed experiments show the authentication confidence level, time required to perform the authentication, and resource consumption by considering a well-known and publicly available smart home dataset [13].

Once the main contributions of this work are presented, it is important to comment that, during the set-up of the PALOT architecture, users establish their identities, which are associated with their behavior patterns. Having the relationship between the pattern and the user’s identity, we are able to provide not only identification but also authentication. In this context, PALOT generates user behavior patterns considering ubiquitous IoT sensors that do not require an authentication process. After that and once users are authenticated, our solution is generic enough to grant or not the access to other kinds of devices such as smart locks, personal computers, or even services, as we further explain throughout the manuscript.

The remainder of the paper is organized as follows. Section 2 gives an overview of the current state of the art of the continuous authentication paradigm in IoT scenarios. Section 3 defines the information model for the continuous authentication framework. Section 4 shows the design details of the proposed continuous authentication system. Upon that formalization, Section 5 characterizes the policies used to authenticate and eventually authorize the users within the system. Finally, Section 6 presents the PALOT architecture, while Section 7 discusses its experimental evaluation in terms of authentication confidence and resource consumption. To sum-up and conclude, Section 8 briefly discusses about the outcomes and the potential future works.

## 2. Related Work

Although IoT solutions are growing in number, security features surrounding “smart objects” remain questionable [14]. The vast amount of information flowing among IoT devices attracts malicious entities aiming to gain unauthorized access to unprotected or insufficiently protected data [15]. Furthermore, traditional attacks such as eavesdropping, man-in-the-middle (MITM), jamming, etc., are still posing a serious threat to the wireless communications between the devices and the infrastructure [16], having the research community to struggle in finding efficient solutions that are able to address these well-known security challenges. This evidence is reflected in the literature where quite a number of works has been proposed to provide effective authentication methodologies for the IoT ecosystem. Beyond traditional encryption-based authentication techniques [17,18], the methodologies that continuously authenticate users and/or devices within the IoT framework look promising since they passively leverage the continuous data flow to achieve authentication duties. In the following, the major proposals are analyzed and grouped into two main categories: *IoT Machine-to-Machine (M2M) continuous authentication* and *IoT User-to-Machine (U2M) continuous authentication*.

### 2.1. IoT M2M Continuous Authentication

With regard to the Machine-to-Machine (M2M) continuous authentication in IoT, researchers and scientific communities have focused on how to provide lightweight authentication primitives when using resource-constrained IoT devices due to their great limitations in computational power. A comprehensive study on authentication and authorization mechanisms for the IoT was presented in [19], and especially oriented to the Industrial IoT (IIoT) in [20]. In the latter, considering that M2M interactions have a greater relevance in the industrial domain, the authors presented seven mechanisms for proximity based authentication depending on specific characteristics inherent to wired, radio, acoustic, light, image, gesture, and biometric methods. These mechanisms were defined to protect the sensory system when taking into account IIoT devices from illicit control, information theft, and service disruptions. Proximity authentication in the IIoT is based primarily on the closeness between devices assuming that interactions are legitimate because IIoT devices are within a certain distance. This assumption, however, could be unjustifiable if one considers that such devices could misbehave because they have been previously compromised by malicious third parties. Another thorough study on authentication protocols for IoT was presented in [21], where more than forty protocols were examined in detail according to the target environment, for example, M2M communications, Internet of Vehicles (IoV), Internet of Energy (IoE), and Internet of Sensors (IoS). Additionally, thirty-five potential cyber-attacks are introduced in [21], defining the underlying threat models and defense protocols oriented to the four previous environments.

A lightweight continuous authentication protocol applicable to a variety of IoT environments was introduced in [5]. By applying hash functions and XOR primitives, the proposed protocol achieves mutual authentication of IoT devices using light-computational operations. More specifically, the protocol utilizes tokens to support the continuous authentication mode in which the tokens contain the dynamic features calculated from the correspondent devices. In this very context, in [22], a secure and efficient authentication protocol making use of a secret sharing scheme was featured. It protects frequent transmissions between IoT devices in short session time intervals in which the secret is agreed upon between the devices during the initial authentication phase and secret actions are utilized as authentication tokens. On the other hand, a secure lightweight authentication and key exchange protocol for IoT devices in a smart home scenario was outlined in [23]. The proposed protocol provides mutual authentication between the IoT devices using a cumulative keyed-hash chain scheme, where security features are demonstrated in a smart home environment with different types of IoT sensors (mainly coffeemaker, bed, air conditioner, camera, and door sensors).

The above-mentioned works have revealed that authentication in M2M communications have mostly focused on lightweight solutions, so that authentication and authorization procedures can be conducted on resource-constrained IoT devices. However, continuous authentication in this context is based on IoT devices that remain authenticated without taking into account changes in their behavior, which in turn could cause upcoming authentication processes not to be carried out.

### 2.2. IoT U2M Continuous Authentication

The User-to-Machine (U2M) authentication in an IoT ecosystem opens up new opportunities and challenges as opposed to M2M authentication processes. Human activities, motions, and (in general) personal, family, and even social behavior make it possible to recognize (authenticate) users in real time by using IoT devices. Ambient Intelligence (AmI) environments that surround humans in everyday life leverage the IoT to deliver advanced added-value services in intelligent scenarios such as smart cities, smart homes, smart offices, and intelligent office buildings among others [24].

Activity recognition has been widely identified in today’s literature as human activity trackers for continuous authentication of users in IoT scenarios. Many works make use of IoT devices such as *smart watches*, providing gesture interaction and a permanent monitoring of physical activities [25]; *wrist-worn devices* for extraction of raw accelerometer data to recognize walking, standing, sitting, and lying activities [26]; *smartphones* to identify the owner periodically by using machine learning (ML) techniques based on anomaly detection for an adaptive continuous authentication system [27]; and *wearable glasses* to discriminate the real owner of the smart object from a potential impersonator using biometric features taken from touch gestures and voice commands [28]. Experiments of this last work, conducted using Google Glasses, showed above 93% detection rate using the collected features. Another proposal is presented in [29], where a transparent authentication system using *brainwaves* as bio-features for IoT networks was proposed. Extracting long-term memory ability from users’ brainwaves, the authors collected the bio-features identified in brainwaves as authentication tokens to perform continuous identification in the background transparently.

The above-mentioned devices are characterized by maintaining a permanent physical contact with humans (e.g., wearables such as smart watches and fitness trackers), but also with a fairly common personal use such as smartphones. However, other devices that do not have direct contact with humans could also be used for recognizing users’ behavioral patterns such as intelligent thermostats and occupancy sensors. In [30], for example, Wi-Fi signals are additionally used to recognize a walking human subject by analyzing Gait patterns. This proposal is based on the fact that Wi-Fi signals reflected by human bodies are generating unique variations in the channel state information measurements. Another important aspect in activity recognition, which has not been widely covered in the literature, is that many works assume that only one user is performing activities within the system. Recently, in [31], the authors proposed a multi-resident activity recognition system for smart homes using a knowledge-driven sequential pattern mining solution. The data-driven solution proposed by the authors are based on statistical and probabilistic theories, in particular on Hidden Markov Model (HMM) where events denoting user’s activities are modeled as a *Markov chain*. Formally, a Markov chain is defined as a stochastic model that describes a sequence of (possible) events whose probability of occurrence of a particular event depends on the state attained in the previous event [32].

In recent years, contextual information retrieved from the IoT devices is also taking on special relevance [33] to strengthen the continuous authentication procedures. In this sense, in [34], the authors leverage the contextual information, obtained from environmental and mobility attributes, to continuously authenticate the users in a smart home scenario oriented toward energy utilization management. Location and tasks’ criticality nature are used as contextual information to select the authentication attributes. User profiles are then dynamically updated over time, thus guaranteeing the adaptability of the entire procedure. Another continuous authentication framework was detailed in [35], integrating contextual information for user authentication in smart homes. The framework proposed by the authors models behavioral profiles based on how users behave or what they do.

With the aim of testing and assessing the solutions analyzed above, authors usually use preexisting datasets that comprise different types of sensors, scenarios of user behaviors and activities performed by users in daily living. In [13], for example, a dataset was produced by the authors with events performed by 24 individuals who performed five different activities in a smart apartment testbed (telephone use, hand washing in the kitchen sink, meal preparation, eating and medication use, and cleaning), collecting the events from digital motion and temperature sensors as well as from analog sensors monitoring water and stove burner use. The collected activities and events are labeled in the dataset depending on the sensor data that was gathered by the environment during the activity execution. Further details of this dataset are provided in Section 7.1. The authors of [13] also presented the implementation of a naïve Bayesian classifier and a Markov model to recognize the five activities commented above. It is worth mentioning that several well-known repositories containing AmI datasets, largely developed by the authors of [13], can be found at the Center for Advanced Studies in Adaptive Systems (CASAS) website [36].

Another interesting dataset was presented in [37], where the authors described a complete dataset produced from 14 sensors that monitored nine different users’ activities. These activities are grouped into three different types or classes: *single* activities that are isolated from the execution of others; *interleave* activities that are performed at the same time than others; and in a *multioccupancy* scenario where activities are being carried out by several people simultaneously. Finally, a recent study presented in [38] deserves to be highlighted, where the authors reported a comprehensive list of existing datasets for human activity recognition as well as a complete study of ML and data mining techniques for activity recognition.

Although the aforementioned works constitute a substantial progress, very few consider a real scenario in which users do not often possess any particular IoT device (e.g., biometric readers and smart glasses). To this extent, a system that is able to correlate the data coming from the IoT devices to authenticate the users in a transparent, passive, and non-invasive manner is missing. This research paper is intended to fill this gap by presenting an autonomous system in which users can be authenticated based on their interactions with the IoT devices.

## 3. Information System Model for Continuous Authentication in IoT

This section presents some definitions and outlines the information system model of PALOT, which has been subsequently defined as an ontology to shape the different components of the proposed continuous authentication system.

### 3.1. System Model

**Figure 1:**
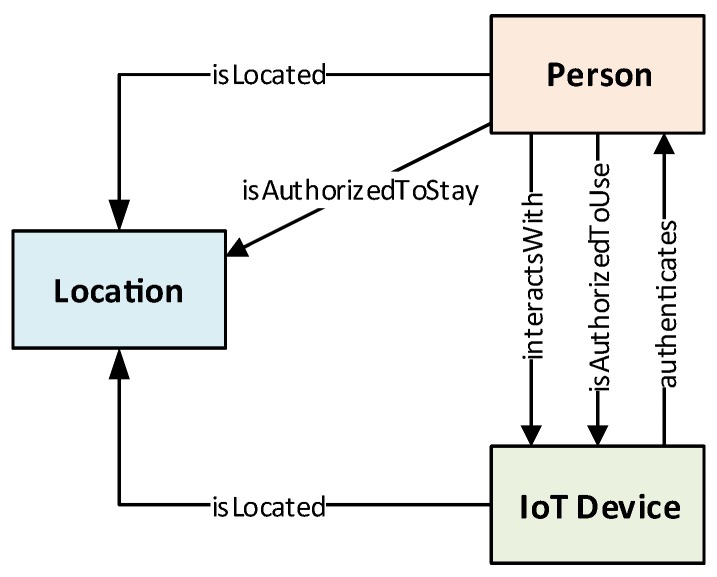
Main components for continuous authentication in IoT.

The components describing our PALOT framework can be found in Figure 1, which is composed of a vector of three main elements defined as PALOT=(D,P,L). In Figure 1, we also observe the relationships among such components, in which the IoT Device component makes reference to the set *D*, Person to *P*, and Location to the *L* set. The definition and modeling of each set is described below.

The main element in any kind of IoT ecosystem is the set of devices shaping the scenario and their features to provide certain services to users and also the system itself. They can be defined as a set of *l* IoT devices (see Figure 1), denoted by $D=\{D_{1},D_{2},\ldots ,D_{l}\}$, where *l* is usually a high number of “thin” devices, which could be, to name a few:

I security devices, willing to support protection;
II sensing devices to acquire given information for monitoring and detecting events of interest; and
III leisure devices enjoyed by users in their daily lives.

These IoT devices will provide different services depending on their functionalities, for example, a service such as a surveillance camera from which to capture videos and images of the environment, or a mail service that users can consume through their smartphones, tablets, or PCs.

Many of the IoT devices are directly utilized by users interacting for working or leisure purposes, while others are deployed in the environment to enable security functions (security cameras or proximity devices among others). Each user is represented in Figure 1 as Person, whose terms (user and person) are used in this paper interchangeably. Persons are modeled as $P=\{P_{1},P_{2},\ldots ,P_{m}\}$, where *m* is the number of users of the environment. A person $P_{i}\in P$ represents a user who can interact with IoT devices, whose relationship can be expressed as $D\left(P_{i}\right)=\left\{D_{i_{1}},D_{i_{2}},\ldots ,D_{i_{x}}\right\}$, although there may also be users who could be authenticated in a continuous way to grant them certain permissions; for example, to stay in a certain area of the environment.

Location also constitutes an important element that needs to be modeled, since the continuous authentication system should consider location-based information in its decision-making processes. Depending on where users are, or the IoT devices’ placement, decision-making processes could grant or deny the corresponding request. Figure 1 depicts certain links between Location and Person and IoT Device in order to trace both users and devices, respectively, within the managed environment. In this sense, Location is modeled as $L=\{L_{1},L_{2},\ldots ,L_{n}\}$, where *n* is the number of places such as areas, rooms, etc., where the user can be found and, therefore, authenticated. Given that users and IoT devices expose a tight relationship with their respective locations, to know in which part of the environment they are located, such locations are modeled as $L\left(P_{k}\right)=\left\{L_{k_{1}},L_{k_{2}},\ldots ,L_{k_{z}}\right\}$ and $L\left(D_{j}\right)=\left\{L_{j_{1}},L_{j_{2}},\ldots ,L_{j_{y}}\right\}$, respectively. For the sake of simplicity, this subsection presents only the model of a reduced number of relationships between components, which is presented in detail in Section 3.2.

It is worth noting that the system model introduced in this section, defining the main components shown in Figure 1, needs to be extended with finer granularity in order to characterize them in more specific sub-components. For example, the IoT devices in *D* should be defined in more detail depending on their functions and features, such as security devices, sensing devices, leisure devices, etc., as mentioned above.

### 3.2. Ontology

Based on the modeling of the main components of the system described above, a collection of ontologies has been created to enable sharing knowledge between the three main components (see Figure 1) and the use of semantic reasoning procedures to infer new knowledge according to the information gathered by the IoT devices.

**Figure 2:**
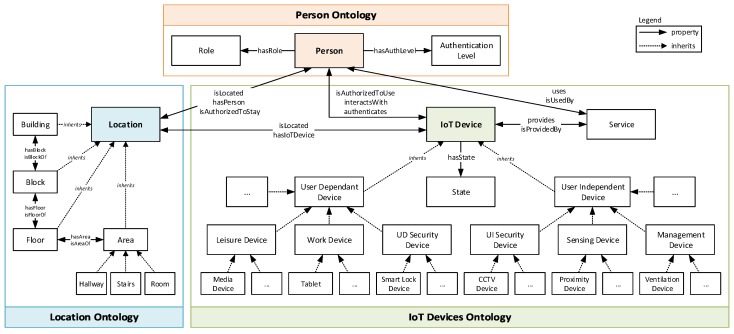
Set of ontologies making up the continuous authentication framework: Location, Person, and IoT Devices ontologies.

Figure 2 shows the collection of ontologies designed, developed, and managed by PALOT (the top-level class within each ontology is depicted following the same colors used in Figure 1), which formally shapes the three main concepts handled by the framework:

- *Location Ontology* modeling a given smart space, which is structured, for example, in different parts of a smart office or home;
- *Person Ontology* to represent any user who is under the framework domains and gets the benefits it offers when using the IoT Devices; and
- *IoT Devices Ontology*, which constitutes the central model of the architecture, as it is composed by the devices providing information about the scenario status and the ones that will apply the reactions decided by the framework to grant or deny actions to users.

The Location Ontology is defined through several subclasses to refine the different spaces in which a given environment is structured, where *Location* is the top-level class from which the rest of subclasses inherit. As shown in Figure 2, this ontology uses a hierarchical model for shaping location, using four subclasses with different levels of size and detail, namely (from the largest to the smallest): *Building*, *Block*, *Floor*, and *Area*. The latter has in turn been divided into more specific spaces: *Hallway*, *Stairs*, and *Room*. The location hierarchical model has been modeled taking into account that our system is oriented to smart offices and smart homes, so that other structures in space could have been modeled considering other location-related subclasses. It is worth mentioning that, in the context of this work, we refer to location as for *symbolic location*, i.e., the place where the user is located, contrary to the physical location which is expressed in coordinates.

The Location class of the Location Ontology is linked to the top-level class of the other two ontologies through several properties. These properties determine the location in which a Person or an IoT Device is located, by making use of the *isLocated* property. In addition, the *isAuthorizedToStay* property has also been defined to determine whether a given Person can be in a certain Location. Here, it is important to highlight that this property is one of the consequences of the authorization policies defined in Section 5).

The top-level class in the Person Ontology is *Person*, exposing two different properties (*hasRole* and *hasAuthLevel* with the *Role* and *Authentication Level* classes, respectively) aiming to find out whether the Person has sufficient roles and authentication levels (i) to use and interact with a given IoT Device (*isAuthorizedToUse* property with the *IoT Device* class) and (ii) to be located in a certain location (*isAuthorizedToStay* property with the *Location* class). These last two properties are the consequences of the authorization policies described in Section 5.2, which are created at runtime by the framework administrator.

Finally, *IoT Device* is the top-level class of the IoT Devices Ontology, which is categorized into two main subclasses that inherit from such root class. In particular, IoT Devices have been modeled depending on whether they are user-dependent devices or not; that is to say, devices with which users interact (e.g., tablets or smart lock devices requiring the user’s fingerprint) or devices deployed in the environment infrastructure (with which users do not interact) to report information that will be further analyzed (e.g., surveillance cameras or proximity devices). Furthermore, the *State* class models the situation of IoT Devices in a given moment of time. Among the possible states, we highlight some of them such as active, interacting, authenticated, in standby, or switched on/off. On the other hand, the *Service* class presents two links to define the different services that a given user could consume (relationship with Person) and to establish the list of services that are provided by such devices (relationship with the IoT Device class).

It has to be noted that the above-mentioned collection of ontologies should be considered generic, since they may be applied to different IoT scenarios. Then, as shown in Section 7.1, the gathered knowledge was tested by leveraging the labeled dataset proposed in [13].

## 4. Confidence Manager

One of the main novel contributions of this research article is the introduction of the confidence manager, whose scope is to recognize users’ behavioral patterns from series of events. Therefore, the essential hypothesis of this research is that each user acts consistently different from the others and this heterogeneity is identifiable by detecting behavioral patterns in the sequence of events gathered by the sensors. Thus, the confidence manager module deployed within the PALOT architecture takes the events collected by the IoT devices and, based on the similarity of the behavioral patterns, it returns the probability that such events belong to a certain user. More specifically, it is able to provide a confidence score related to the above-mentioned similarity in real-time, i.e., while the users are performing daily activities.

To formalize such user’s behavioral patterns, it is mandatory to introduce the following definitions.

**Definition** **1** (Event)**.** *An event is a message generated either periodically (e.g., temperature) or upon status changes (e.g., door open) by any IoT device connected to the smart environment and modeled by the defined ontology.*

**Definition** **2** (Activity)**.** *An activity is defined as a series of events (activity track) belonging to a specific pool of sensors in a time frame.*

To provide an example, the activity track of *enter the building* may be composed of events potentially generated by a door sensor, proximity sensors, movement sensors, light-switch sensors, microphones and cameras. It is clear that the sequence of these possible events depends on the state achieved by the previous one.

For example, if the door is open, the user *most likely will* close it before leaving the hall. One might say that the full history of events influences the probability of the next one , demonstrating that the sequence of possible events can be assumed and modeled as a Markov chain as formally defined in [32] (i.e., within the activity, the future event only depends on the state of the current one, and not on the sequence of previous events). A process with this characteristic is called a Markov process. It is worth mentioning that each activity can have a different time duration and the actions of several persons (or one doing multi-tasking) are considered as different activities.

As a result, the main component of the confidence manager is a behavior profiler that can be instantiated and trained for each user registered in the ontology previously described (Section 3.2). As for the profiler, Algorithm 1 provides the formal definition of the training process, while Algorithm 2 presents the testing one.

The machine learning driver of the proposed confidence manager module is a series of Markov chains. To be more precise, for each user included in the dataset, a transition matrix is trained using the information obtained from the activity tracks. The prediction process is performed by calculating the probability that the examined chain of events belongs to any of the trained users.
**Algorithm 1** Profiler Training—TRAIN
$\left(S,E,u\right)$.**Require:**S ≠ null               ▹ All possible sensors’ events**Require:**|E|>1                 ▹ List of events for the user *u***Require:**u ≠ null                     ▹ User’s identifier **for all** s∈S **do**          ▹ Initialize the Markov transition matrix *T*   **for all** r∈S **do**     $T\left[s,r\right]=0$   **end for** **end for** $e_{c}$ = First(E)                  ▹$e_{c}$ is the current event **while** E has next **do**   $e_{n}$ = Next(E)                    ▹$e_{n}$ is the next event   $T[e_{c},e_{n}]+=1$        ▹ Count the occurrences of the events’ pairs   $e_{c}=e_{n}$          ▹ Update the current event, i.e., move forward **end while** **for all** row *r* of *T* **do**   T[r] = Normalize(T[r])            ▹ Normalize each row **end for**
**Algorithm 2** Profiler Testing—TEST
$\left(e_{c},E\right)$**Require:**$e_{c}$ ≠ null                           ▹ Current event**Require:**|E|>1                            ▹ List of future events**Require:***T* is initialized                   ▹ The profiler has been trained h=0                                  ▹ Number of hits m=0                               ▹ Number of misses **while** E has next **do**   $e_{p}$ = argmax$T\left[e_{c}\right]$      ▹$e_{p}$ is the predicted event that follows the current one   $e_{n}$ = Next(E)                           ▹$e_{n}$ is the next event   **if** $e_{p}=e_{n}$ **then**     h+=1                ▹ Hit if the next event is correctly predicted   **else**     m+=1              ▹ Miss if the next event is not correctly predicted   **end if**   $e_{c}=e_{n}$                          ▹ Save the current event **end while** **return** $c=\frac{h}{h+m}$                          ▹ Return the confidence

**Figure 3:**
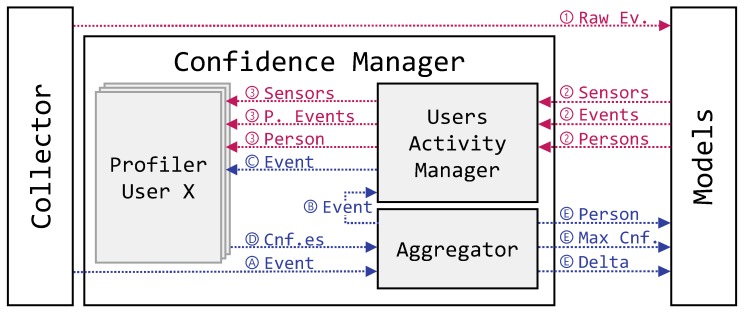
Overview of the PALOT new confidence manager module.

While both processes are carried out in the confidence manager, the training phase requires knowledge about the system that needs to be provided. To begin with the training phase (Algorithm 1), the process is highlighted in Figure 3 with the red color and identified with circled numbers. The training phase, as described below, is triggered manually by the framework administrator and it requires the human interaction for labeling purposes. As previously mentioned, the users that need to be continuously authenticated within the IoT scenario are asked to perform daily activities. In there, the events generated by the interactions with the surrounding IoT devices are collected in order to be processed by the proposed framework PALOT .

Following the control flow, the training phase is composed of four steps here identified:

(1) the collector acts as source, passing the raw training events to the modeling ontology described in Section 3.2;
(2) the events are enhanced with knowledge regarding the users (e.g., the framework administrator manually labels the instances) and the sensors that have been registered in the system and thus reflected to the confidence manager; and
(3) inside the confidence manager, a submodule called “user activity manager” takes care of instantiating the profilers (one for each registered user) with the labeled events plus the registered sensors and initiates the training phase according to Algorithm 1.

Regarding the testing phase, the process is also illustrated in Figure 3 in blue color and identified with circled letters. Specifically, starting from the events gathered by the collector, this phase is able to output the *most probable* user which is performing the related activity together with the confidence score of such decision, in the form of probability, and the absolute difference among the two highest confidence values, to which we refer as *delta*. Thus, the training phase is composed of five steps, here described:

(A) Firstly, the collector acts as source, passing in real-time an unlabeled event to the aggregator.
(B) The aggregator replays the event to the user activity manager.
(C) The user activity manager in turn shares it with all the registered user profilers, looking for similarities in behavioral patterns.
(D) The aggregator collects all the confidence scores calculated by the registered profilers according to Algorithm 2.
(E) Finally, it returns the user with the maximum score, its confidence and the delta, calculated according to Algorithm 3.

**Algorithm 3** Confidence Delta—$\delta \left(P\right)$.**Require:**P ≠ null                               ▹ Registered profilers $u_{max},c_{max}=\left\{u,c\in P:c=\max\left(P.values\left(\right)\right)\right\}$   ▹ Find the user with the maximum confidence $P=\{u,c\in P:u\neq u_{max}\wedge c\neq c_{max}\}$                 ▹ and remove it from the set $\delta =c_{max}-\max\left(P.values\left(\right)\right)$             ▹ Calculate the δ between the two highest values **return** δ                                        ▹ and return it

Note that there are no restrictions regarding the size of the testing events list. The proposed Algorithm 2 works perfectly with a single event, thus enabling real-time authentication capabilities. That is, at this prototypical stage, the recorded events might be analyzed individually or in batches, according to the specific configuration chosen by the framework administrator.

## 5. Policy-Based Decision-Making System

The proposed solution continuously authenticates and authorizes users to stay in certain spaces or utilize different IoT devices by employing semantic rules, which form policies. The proposed architecture uses rules composed of two lists of predicates, the antecedent, and the consequent. If all predicates of the antecedent part take the Boolean value true, all predicates in the consequent part are evaluated. It is important to know that in our semantic rules the predicates in the consequent part establish new relationships between entities of the ontologies, but do not generate new entities.

Our policies are composed of the following elements: *Type* is the kind of policy; *Target* is the person considered by the policy to be authenticated or authorized; *Location* is the place or environment in which the policy is applied; *Confidence* is the output of the confidence manager; and *Result* determines the relationship that the Target will have with the IoT Device or Location regarding authentication and authorization grants. Note that Result is the consequent part of the semantic rule, while the remaining fields belong to the antecedent part [39].

Our system manages two kinds of policies: *Authentication* and *Authorization* policies. Both families are defined by the Framework Administrator to decide the authentication and authorization of the users to stay in a given space or use specific IoT Devices. Below, we show an example for each one of these policies.

### 5.1. Authentication Policies

Authentication policies consider the information modeled by the ontologies as well as the output of the confidence manager, which returns the user with the maximum score, its confidence, and the delta. Different levels of authentication are generated by policies according to the previous fields.

The antecedent of authentication policies considers different elements belonging to the three proposed ontologies (Person, Location, and IoT Devices) and the confidence manager. On the other hand, the consequent part generates relationships between entities belonging to the Person ontology. As an example, the next policy indicates that users will be continuously authenticated with the Green authentication level when they have a confidence level of 0.7 or higher, a delta score of at least 0.6, and their location is the *smart home*. At this point, it is important to note that the confidence level and delta values have been set-up according to the experiments presented in Section 7.

Person(?person)∧
hasConfidence(?person,?confidence)∧greaterThan(?confidence,0,7)∧
hasDelta(?person,?delta)∧greaterThan(?delta,0,6)∧
isLocated(?person,#SmartHome)→
hasAuthLevel(?person,#GreenAuthLevel)

### 5.2. Authorization Policies

Authorization policies take into account the level of authentication, provided by the Authentication policies, to allow users to stay in certain locations or use specific devices located in the users’ environment. By default, the proposed architecture denies the authorization in the absence of rules. As an example, the next policy authorizes users to stay in the *smart office* and utilize its *IoT Devices* when they are located in that room with the *GreenAuthLevel* authentication.

Person(?person)∧
hasAuthLevel(?person,#GreenAuthLevel)∧
isLocated(?person,#SmartOffice)∧
hasIoTDevice(#SmartOffice,?ioTDevice)→
isAuthorizedToUse(?person,?ioTDevice)∧
isAuthorizedToStay(?person,#SmartOffice)

## 6. PALOT Architecture

**Figure 4:**
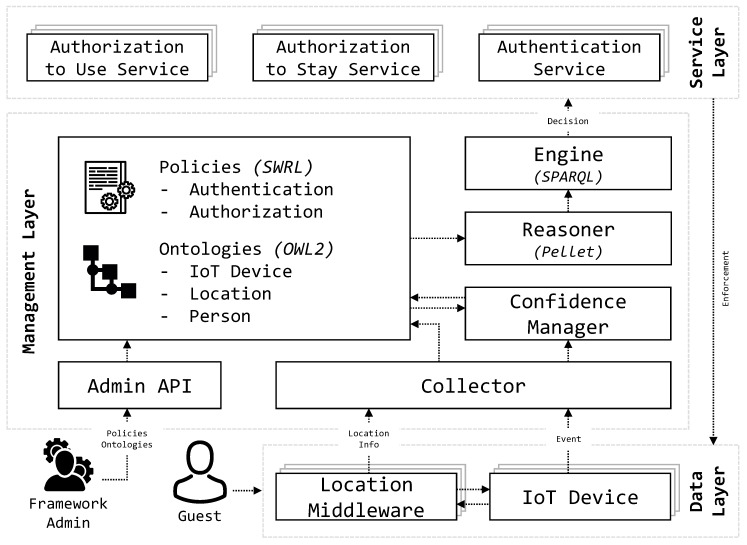
Overview of the PALOT multilayered architecture.

This section shows the proposed architecture, which is able to provide users with a continuous and non-intrusive authentication and authorization solution according to their interaction with heterogeneous IoT devices. To reach the transparent authentication and authorization processes, PALOT encompasses several modules organized in three different layers according to their functionalities. Figure 4 illustrates the *Data*, *Management*, and *Service* Layers composing the presented architecture.

### 6.1. Data Layer

This lower level includes all the *IoT Devices* belonging to the smart-environment. Each device outputs a stream of events according to the device purpose. As defined in Section 4, these events might be generated either periodically (e.g., CCTV camera frames, temperature sensors, etc.) or upon status changes (e.g., interaction with the device, movement sensors, etc.). As modeled by the ontology specified in Section 3.2, there exist two main categories of IoT devices, according to which the events’ stream can be either sporadic or continuous. These events are filtered and all the information that might be used for authentication purposes is sent to the *Collector* module in the Management Layer.

A *Location Middleware* module is taking care of locating all the devices belonging to the framework. For example, a device such as a CCTV camera might not be able to provide a localization service, thus it has to be located through other information, such as the unique identifier and the installation location. As for the *IoT Devices*, the location data are sent to the *Collector* that will interpret them according to the previously mentioned ontology (Section 3.2).

### 6.2. Management Layer

This layer represents the intelligent core of this framework. It provides several features, among them the possibility of modeling all the events generated by the *Data* layer according to a standard format (the ontology). This function is required in order to be able to provide both the authentication and the authorization services responsible for controlling the IoT devices. In this context, these capabilities enable the framework to authenticate users according to their behavior and interaction with the devices, without losing the capabilities of providing layered security. It is clear, in fact, that the layer receives different inputs from multiple devices that are collected, integrated, and analyzed.

Three main sources of knowledge for the *Reasoner* module are found, namely the *Policies* and the *Ontologies* databases and the *Confidence Manager* module. The first one contains the definition of all the policies structured in two groups, one for the authentication (Section 5.1) and one for the authorization (Section 5.2); the second one includes the three ontologies of IoT Device, Location, and Person as described in Section 3.2; and the last one is in charge of learning the users’ behavioral model in order to be able to recognize them as described in Section 4. However, the control flow starts with the location information and events’ streams that are collected by the *Collector* module, which takes care of interpreting and saving them in the aforementioned ontologies database.

The information is then used by the *Reasoner*, whose implementation details are highlighted in Section 7, which ultimately triggers a decision and a reaction in the *Engine*. The decision is taken according to the policies specified in the aforementioned database applied over the collected events modeled upon the class-specific ontology. The decision is hence enforced by retro-fitting the reaction for the *Data* layer. Finally, an administrative API is available for human administrators in order to be able to configure (i.e., add, edit or delete policies and ontologies) and tune (i.e., frequency of update, etc.) the system.

### 6.3. Service Layer

This layer contains the set of services provided by our solution to interact with different IoT devices and authenticate or authorize users to employ specific devices or stay in given places. Here, it is important to highlight that some devices such as boundary authentication gateways do not require these services because they are used firstly to identify users. Thus, our solution has a specific authentication and authorization service for each IoT device. To be able to manage this process, some devices expose APIs that permit the authentication and authorization of the users that are interacting with them. Multiple services can be included later on for increasing the maneuverability and the reactiveness of the system to external inputs [40].

### 6.4. Actors

The main actors included in this architecture are the *Framework Admin*, whose tasks include the configuration and the maintenance of the policies and ontology database, and the *Person* who may interact with the devices. The benefits for persons are clear, since avoiding intrusive authentication and authorization procedures greatly simplifies the user experience without jeopardizing the environment security. In fact, independently from their active interaction, our framework is built so to be able to recognize and profile them, in order to provide real and continuous authentication.

## 7. Deployment and Experimental Results

To deploy, analyze and validate PALOT, we designed several experiments aiming to characterize the framework performances in terms of both resource consumption and confidence of the authentication process. To test cross-compatibility and scalability, experiments were conducted on different machines and, when not otherwise specified, the experiments were run on both devices multiple times, thus the reported metrics are to be considered as the average of all the executions. Here follows the list of machines:

- a Dell M3800 workstation with an Intel i7-4712HQ processor running at 2.30 GHz, 16 GB of DDR3 RAM at 1600 MHz, using Windows 10 (Build 17134) OS; and
- a personal laptop with an Intel Core i7-3770 running at 3.40 GHz, 16 GB of DDR3 RAM at 1600 MHz, using Ubuntu 16.04 LTS OS.

The experiments were designed in three categories, targeting:

I the resource consumption of the decision-making modules (Section 7.2);
II the performances and scalability of the authentication module (Section 7.3); and
III the efficacy and confidence of the authentication module (Section 7.4).

As already mentioned and when not otherwise stated, the experiments were conducted as described in Section 7.1.

### 7.1. Dataset and Motivating Scenario

The dataset presented in [13] and used for the evaluation of our proposed framework includes events for 24 individuals, with a total of 120 activity traces and 6425 events. More specifically, participants were asked to perform certain daily activities (i.e., make a phone call, wash hands, cook, etc.), while interacting with the surrounding sensors within the smart apartment. The sensors used in [13] are a collection of commons IoT sensors that can be found in a smart environment, e.g., sensor to measure motion, temperature, item usage, etc. The collected data are fully labeled, detailing which participant is performing the specific activity. It is worth mentioning that the participant does not perform activities in parallel, that is, each of them carries out the tasks in a sequential fashion.

The proposed system generates the users’ behavior patterns considering IoT ubiquitous sensors (motion, presence, light, or water sensors) that do not require authentication mechanisms. Once we authenticate the user by considering the events generated by the previous IoT devices, our solution is generic enough to grant or not the access to other kinds of devices such as smart locks, personal computers, or even services. In any case, it is also worth noting that PALOT provides different levels of authorization and, according to that level, a given user can perform (or not) particular actions. However, in this work, we consider that IoT devices used to generate behavior patterns do not require authentication mechanisms.

In the previous scenario, during the set-up of the PALOT architecture, users establish their identities, which are associated with their behavior patterns generated during the training phase. After that, during the evaluation phase, for each user the proposed solution is able to identify the most similar behavior pattern. Having the relationship between the pattern and the user’s identity, we are able to provide not only identification but also authentication.

### 7.2. On the Performances of the Decision-Making Modules

We deployed PALOT to validate its proper functioning and measure its throughput and scalability. In this context, the representation of the information (ontologies and policies) and the decision-making process (Reasoner and Engine) are based on Semantic Web techniques, where Location, Person, and IoT Device ontologies, shown in Section 3, are defined in OWL 2 (Web Ontology Language) [41] and were generated with the Protégé tool [42]. We chose OWL 2 rather than other languages such as RDF, RDFS, or DAML+OIL because OWL 2 is more expressive than the rest. It was specifically designed as an ontology language, being an open standard, and the main ontology language used nowadays in Semantic Web. On the other hand, semantic rules defining the policies in Section 5 are expressed in SWRL (Semantic Web Rule Language) [43]. SWRL includes a type of axiom, called *Horn clause logic*, of the form if…then…, being the most widely used solution in Semantic Web today.

The proposed architecture makes decisions about the authentication and authorization of users according to the previous ontologies and semantic rules. For that, a semantic reasoner, implemented by the Reasoner component of Figure 4, infers new knowledge that decides whether a given user is authenticated, authorized, or not. We used Pellet [44] as semantic reasoner, which receives ontological models with the information shaped by the ontologies and policies. Finally, the Engine component of the proposed architecture is in charge of applying periodical queries, performed in SPARQL [45], to the inferred model and gets the result about the authentication and authorization of users.

We conducted several experiments with the aim of measuring its throughput and scalability. These experiments were intended to deal with two questions:

- Is the decision-making process time acceptable?
- How does it scale with different IoT devices and authentication/authorization policies?

It has to be noted that the results shown in this section were obtained by executing the experiments 100 times and computing their arithmetic mean.

A way to measure the performance of the decision-making process is doing executions with different levels of complexity. This complexity is related to the number of individuals present in the ontologies and the number of semantic rules making up the policies. Increasing the number of individuals and semantic rules will provoke an increment on the number of statements, and thus on the complexity of executions. The number of individuals contained in our ontologies is referred as *population*. This was randomly generated, but in a controlled way to achieve a real distribution of the elements composing the environment. Table 1 depicts the number of elements used in our environment and their percentages.

Another important aspect, related to the second question highlighted in this section, is to evaluate the scalability of the decision-making process. With this goal, we defined an initial population of 30,000 individuals, which was increased by 30,000 individuals in each step. Table 2 shows the complexity of the proposed ontologies (relationships between the individuals and the statements generated by the semantic reasoner). As observed, the number of statements is proportionally increased according to the number of individuals.

**Figure 5:**
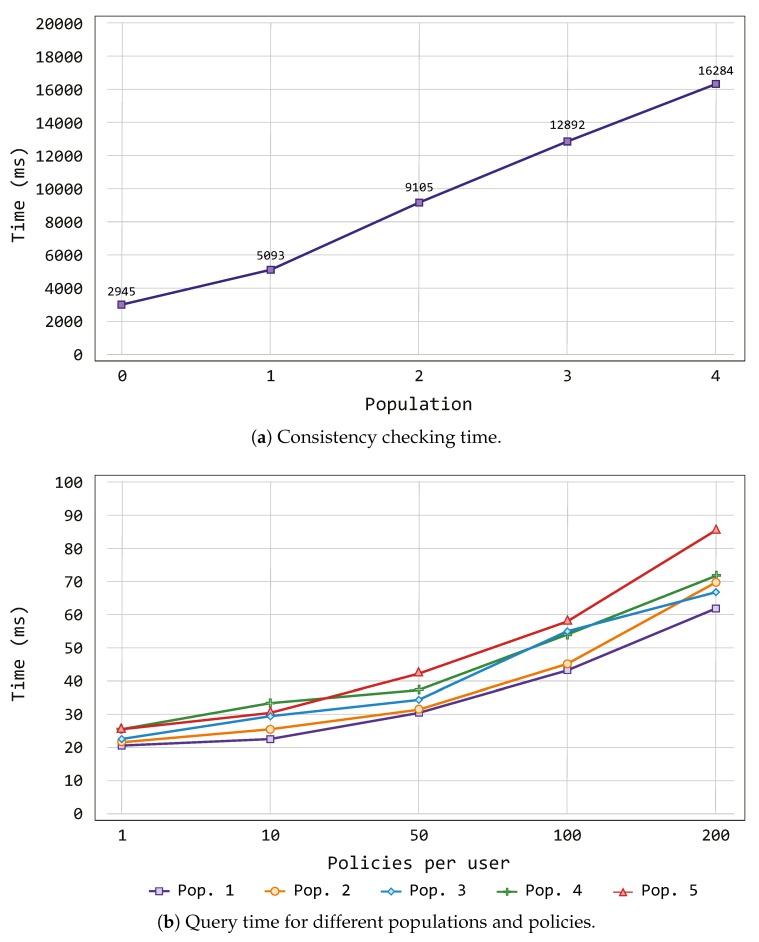
Time required by the PALOT decision modules.

Figure 5a depicts the time, measured in milliseconds (ms), used by the semantic reasoner to validate the ontology considering different population groups (shown in Table 2).

Such time is used by the reasoner in order to infer knowledge starting from the information modeled within the ontologies. The result of this operation is a model which contains each individual of the proposed population, and in this context each individual of the smart scenario. Comparing the increase of individuals and statements with the time required by the decision-making process, we can observe that the proposed solution can support a very large number of individuals or statements within a reasonable time. Furthermore, the linearity property behind these results allows us to deduce that a better computer system setting would obtain lower reasoning times.

The above experiment demonstrated a linear relationship between individual/statements and the reasoning time, but without considering policies. Thus, the main goal behind the next test was to check how policies affect the scalability of the proposed solution. In this sense, we defined several percentages of policies related with the persons contained in our population groups.

Figure 5b shows the variation of the time required by the Engine component when it makes a query for different populations (see Table 2) and numbers of policies. As previously mentioned, such time is needed to get the result about the authentication and authorization processes based on the policies. Thus, the process of querying the formerly generated model must be executed following the flow of events stemming from the IoT devices within the smart scenario. As we can see, policies have a very low impact in our framework. For all populations, the difference between having 10 and 200 policies per user is around a few milliseconds.

As main conclusion of this section, we demonstrated with the previous experiments that, when the number of individuals/statements is linearly increased in our ontology, the decision-making process time also increases linearly. Furthermore, the semantic rules that form the policies do not have an important impact on the decision-making process time.

### 7.3. On the Scalability of the Authentication Module

Undoubtedly, the scalability of the system while performing the continuous authentication process represents a critical factor. On the one side, a low impact on the resource consumption is highly desirable, especially when considering scenarios in which the computational power is limited. On the other side, the overall time required to authenticate the users has to be reduced as much as possible to accommodate the real-time events stemming from the smart environment. Bearing in mind such consideration, we stressed the proposed authentication component to discuss on its scalability in terms of resources (Experiment 1) and time (Experiment 2), as reported in the next sections.

**Experiment** **1 (CPU and RAM consumption).** *Identify the impact of the Authentication module on the resources.*

**Experiment** **2 (Execution time).** *Measure the time elapsed to perform Training and Testing procedures.*

#### 7.3.1. CPU and RAM Consumption

**Figure 6:**
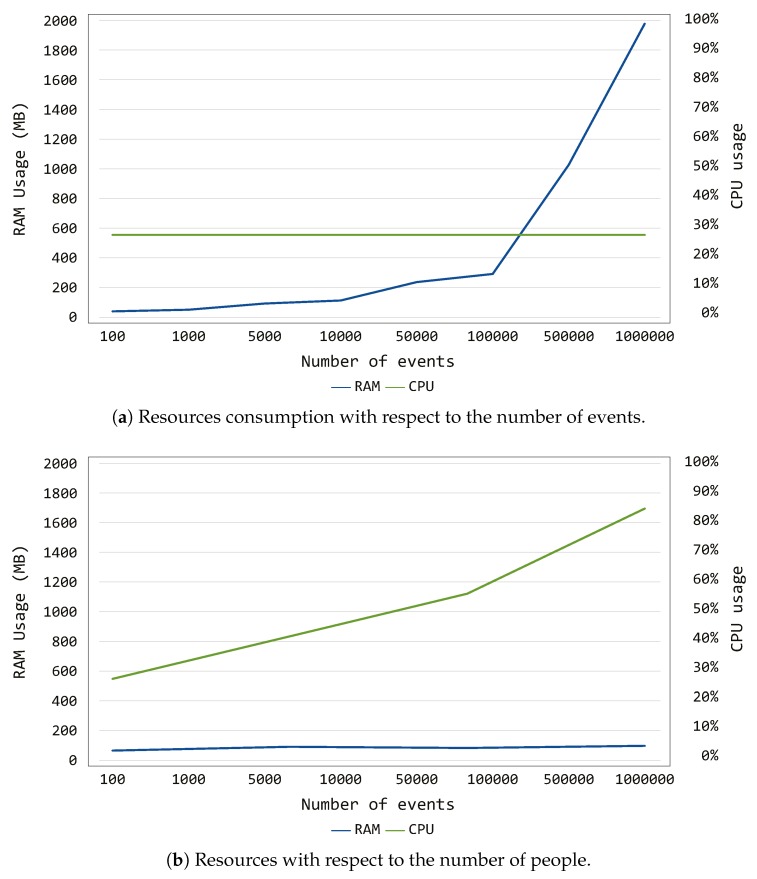
Resource consumption of the proposed framework with respect to number of people and events.

To argue on the resources consumption of the proposed Continuous Authentication framework, several experiments were conducted aiming at stressing it. Specifically, while the system was performing the continuous authentication duties, CPU and RAM consumption were monitored, as illustrated in Figure 6. In the figure, the arithmetic average is plotted to avoid possible spikes and outliers. More in detail, we decided to carry out two experimental sessions:

1. resources consumption (in terms of CPU and RAM usage) while increasing the number of events belonging to the targeted users, as depicted in Figure 6a; and
2. resources consumption (in terms of CPU and RAM usage) while increasing the number of targeted users, as shown in Figure 6b.

Regarding the first experiment, CPU and RAM were monitored while increasing the events belonging to the target users from 100 to 1 million. As depicted in Figure 6a, the proposed system is not particularly CPU-demanding, since its utilization remained at 30% steadily during the entire experiment. On the contrary, the RAM usage presents an increasing linear trend, reaching 2 GB to analyze 1 million events. Such results can be explained by looking at the operations performed by the Continuous Authentication system: by increasing the number of events, the data structures needed to store the behavioral patterns of the target users become wider. Thus, the more events are used to train the model, the more RAM memory is used. Nonetheless, one could say that the number of events the proposed framework has to analyze during its normal operations does not reach such high value (i.e., 1 million). Moreover, the RAM usage for 100,000 events is lower than 400 MB, which can be considered as acceptable for a quite large events sample.

Regarding the second experiment, CPU and RAM were monitored while increasing the number of targeted users the system is continuously authenticating from 10 to 500. As shown in Figure 6b, in this case, the RAM required during the experiment is quite constant, remaining at ~120 MB of utilization. On the other side, the CPU usage reaches 80% while the system is performing the authentication process for 500 people. Such behavior can be justified by considering that the system requires more cycles when processing more targeted users. This characteristic directly implies that, while the RAM utilization remains quite constant, the CPU shows a trend with linear increment when increasing the number of users.

The conducted experiments on the resources consumption of the Continuous Authentication framework showed that the proposed system presents a RAM-demanding trend when the number of considered events become huge, while it shows a CPU-demanding tendency when increasing the number of users. Nevertheless, the framework performs well in normal conditions: it utilizes less than 400 MB to analyze 100,000 events, and it uses less than 60% to authenticate 100 people. Such results demonstrate its applicability to smart environments, such as smart home or smart office.

#### 7.3.2. Execution Time

Among the desirable characteristics of a continuous authentication system, it is clear that the time required to perform the authentication duties is among the most critical factors. In fact, the system must be able to analyze the event stemming from several smart objects in an acceptable time in order to grant the authentication to the users within the smart environment. With this mindset, we conducted a set of experiments to test the execution time of the proposed system. In particular, we registered training and testing times separately while increasing the number of incoming events.

**Figure 7:**
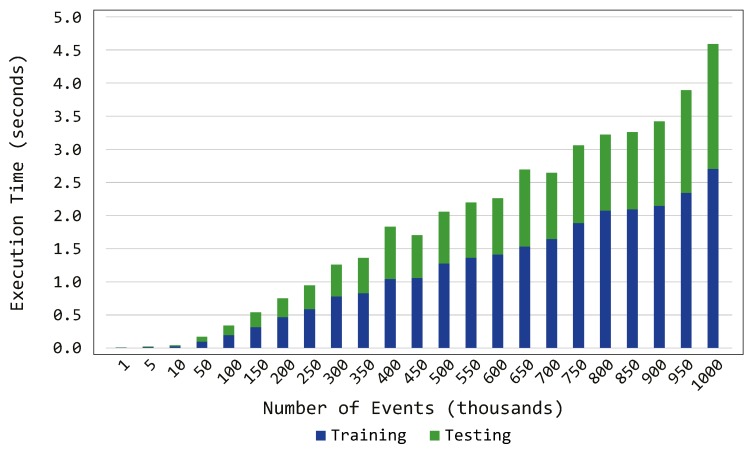
Training and testing times of the proposed system.

Figure 7 shows the relation between the execution time and the number of events. As expected, the system requires more time to train the model than to perform the testing process. In fact, during the training phase, the probabilistic model is created, while, during the testing phase, the events are directly compared against such model. Another interesting feature is the increasing trend shown by the execution time: that is, when increasing the number of events, the time required by the system linearly increases, reaching ~4 s to deal with 1 million events. Such result allows one to argue that the required execution time scales accordingly with the number of incoming events, thus demonstrating the scalability of the presented component. In addition, the time required to train the model with 100,000 events, and to test 100,000 events against such generated model, is less than 0.5 s, which seems more than acceptable in the context of the proposed scenarios (i.e., smart home or smart office).

### 7.4. On the Efficacy of the Authentication Module

As described in Section 4, the proposed framework works by comparing the predictions of all the users registered in order to find the right one. To evaluate such approach, two experiments were defined, aiming to study and analyze the evolution of the confidence in the authentication (Experiment 3) and its adaptation to the dataset used as source (Experiment 4).

**Experiment** **3 (Confidence evolution).**  

*Training—A set of activity tracks belonging to the Target User.*

*Testing—A set of activity tracks belonging to all the 24 other users.*

*First Task—Identify in the testing set the activity tracks belonging to the Target User.*

*Second Task—For each activity track in the testing set, establish the confidence of the classification as defined in Section 4.*

**Experiment** **4 (Dataset evolution).** *Measure possible improvements based on the dataset enhancement.*

#### 7.4.1. Confidence Evolution

As previously mentioned, Experiment 3 was designed to analyze the feasibility of the authentication process. It is composed of 24 parts, and, to be more precise, the chosen dataset [13] features 24 guests performing everyday activities in a smart environment; the application scenario and the dataset are introduced in Section 7.1. The PALOT approach is capable of recognizing the guest after a few sensor events.

**Figure 8:**
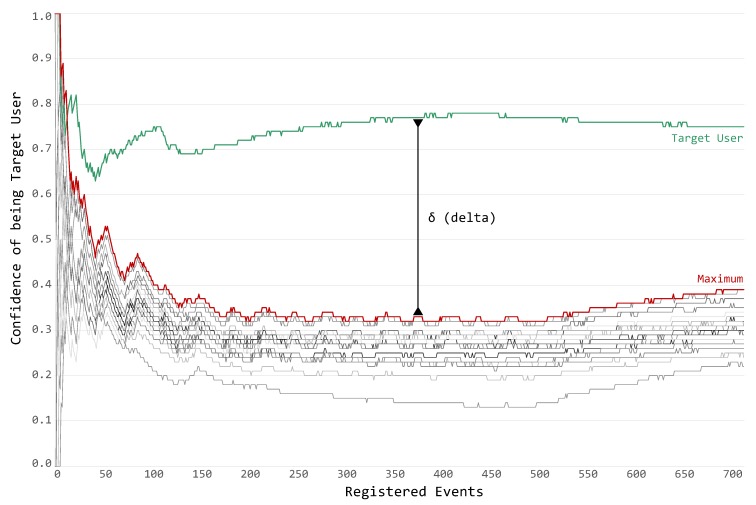
Confidence evolution for a target user.

Figure 8 presents the evolution of the confidence for an example guest (i.e., user P23) with respect to the number of sensor events over a long time period (multiple activity tracks). On horizontal axis, multiple activity tracks are combined, highlighting the number of sensor events. On the vertical axis, the confidence score (calculated as defined by Algorithm 2) for the Target User (colored in green), and all the other users (colored with different shades of gray) is presented. Moreover, to help identify the previously defined *δ (delta) score* (Algorithm 3), both the maximum and the δ itself have been plotted.

By walking through the chart, from left to right, it is possible to notice:

- the *bootstrap* phase (between zero and 35 events), in which the system has seen too few events to make a proper prediction (i.e., the *delta score* is very small and abruptly changes);
- the *wavering* phase (between 35 and 50 events), in which the system has already a good hint about the classification result, but not a final decision (i.e., the *delta score* is lower than the designed *threshold*: δ<θ); and
- the *authentication* phase (after 50 events), in which the system is confident enough to properly make a decision regarding the Target User.

**Figure 9:**
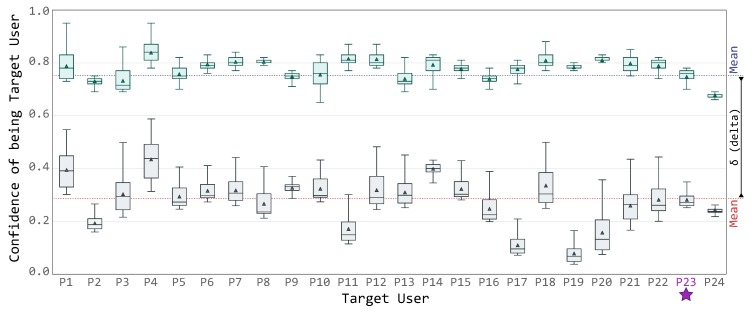
Confidence evolution across the dataset.

While Figure 8 presents details about a specific Target User, Figure 9 presents how these distributions vary across the whole dataset during the *decision* phase. Specifically, both figures shares the vertical axis, i.e., the confidence score for the specific Target User, while the latter figure presents each guest’s view on the horizontal axis.

Figure 9 is divided in three parts:

- the green box plots, representing for each user its target confidence distribution, i.e., the green series presented in Figure 8;
- the gray box plots, representing the average confidence distribution of all the other users, i.e., the gray series presented in Figure 8; and
- the Target User average confidence score represented as a dotted blue line, i.e., the mean of the average values of all users’ confidence scores; and, symmetrically, the other users average confidence score represented as a dotted red line, i.e., the mean of the average values of all other users series.

As shown in the boxplot chart in Figure 9, by using the confidence delta (as specified in Algorithm 3), it is possible to clearly recognize the targeted user even when the confidence scores of the other profiles are quite high (e.g., user P4). To be more precise, the confidence score of each targeted uses varies between 0.63 (minimum of user P24) and 0.98 (maximum of user P4), with an average value of 0.78. On the contrary, the other users’ profiles achieves results as low as 0.08 (minimum of user P19) and as high as 0.59 (maximum of user P4), with an average of 0.29.

It is up to the framework administrator to define the minimum threshold for the delta score, to minimize the effects of classification errors and mimicking attacks.

#### 7.4.2. Dataset Evolution

**Figure 10:**
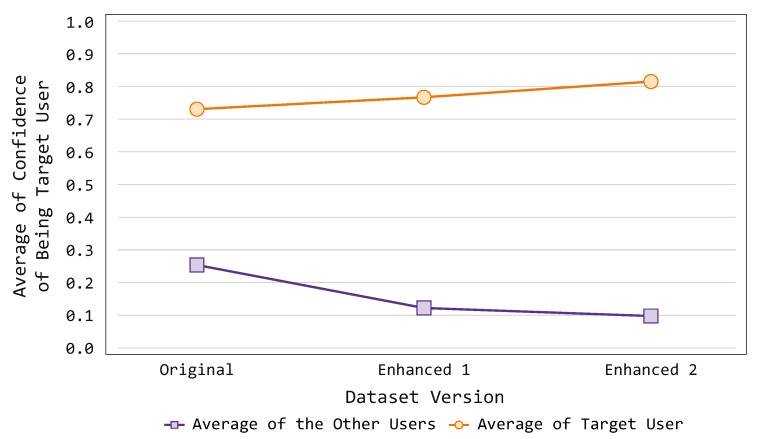
Confidence with regards to the quality of the dataset.

As already mentioned, the proposed PALOT can seamlessly authenticate and authorize the user to perform certain activities within the designed scenario. The authentication process has been proved to be reliable during the experimental sessions on the tested dataset [13] (as previously described in Section 7.1), reaching a more than acceptable confidence level. Specifically, the system is able to recognize the targeted users with a confidence level above 70%, and it can also distinguish among different users. It is worth mentioning that the dataset used during our experiments was initially created with the aim of recognizing the activities performed by the users within the smart environment. Thus, we leveraged such data to recognize the behavior of a specific person who was carrying out the activities. In this context, one could say that the data provided by the dataset are quite poor (a few hundred events per person). Hence, we decided to test the authentication capabilities of the proposed system when repeating some of the activities of the targeted users (Experiment 4). Figure 10 illustrates the results of such experiment, comparing the confidence level with different versions of the dataset, which are explained as follows:

- Original: The original version of the dataset, from which we extract the labeled activities.
- Enhanced 1: A second version of the dataset, in which we replicated 10 activities for each user.
- Enhanced 2: A third version of the dataset, in which we replicated 20 activities for each user.

In particular, we plotted the confidence level our authentication system provides for each version of the dataset. It is possible to notice that the confidence level regarding the targeted users (i.e., the capability of the system of recognizing person) is increasing when considering the different versions. Specifically, it rises above 80% with the last proposed version. On the other side, it also notable that the confidence level with regards to the other users (i.e., the capacity of the system of distinguish person) is decreasing throughout the experiment. In fact, it drops below 20% when considering the dataset with 20 activities repetitions. Such results allow one to claim that the overall authentication performance of the proposed system may further improve when the quality of the analyzed dataset (in terms of number of events) enhances.

## 8. Conclusions and Future Work

Despite the numerous efforts to develop successful authentication systems, a number of challenges remain unsolved. In particular, most of the times the quality of experience of the users tends to be low, since they have to carry intrusive devices or remember complex authentication secrets. In this regard, the main contribution of this paper is a novel IoT-enabled continuous authentication framework called PALOT. Specifically, we designed and developed an ontology to formally model IoT scenarios and designed an architecture to deal with the current status of a given IoT scenario. Within such architecture, we introduced and integrated a novel module called “confidence manager”, whose main task is to recognize users’ behavioral patterns by leveraging the powerful capabilities of the Markov models. The output of the confidence manager, together with the other elements of the ontology, is then used to authenticate and authorize users by employing semantic rules, which form policies.

Within our framework, users are seamlessly authenticated and authorized without requiring any additional device. However, these processes are natively dependent on the deployment context, thus requiring extensive training to correctly model the users’ behavior. To demonstrate the suitability of the proposed solution, we designed several experiments aiming to characterize the framework performances in terms of both resource consumption and confidence of the authentication process.

As future work, we intend to apply further machine learning techniques to possibly argue on the pros and cons of each methodology. Additionally, we plan to evaluate PALOT while multiple users interact with each other, thus extending the above-mentioned concepts of event and activity. Finally, we also plan to perform adversarial attacks to measure how easy or difficult it is mimic the behavior of particular persons.

## Figures and Tables

**Table 1:** Individual distribution of population.

Element	Amount	Percentage		Element	Amount	Percentage
Buildings	1	0.1%		Persons	4	0.1%
Floors	4	0.2%		Roles	10	0.3%
Areas	20	0.6%		IoTDevices	1000	31.0%
Sections	80	2.5%		Others	100	3.1%
Positions	2000	62.1%		**Total**	**3219**	**100%**

**Table 2:** Number of individuals and statements per population.

Population	0	1	2	3	4
Individuals	30,000	60,000	90,000	120,000	150,000
Statements	352,532	710,004	1,065,537	1,465,409	1,804,336
